# MicroRNA-155 Protects Group 2 Innate Lymphoid Cells From Apoptosis to Promote Type-2 Immunity

**DOI:** 10.3389/fimmu.2018.02232

**Published:** 2018-10-09

**Authors:** Martin D. Knolle, Shau Bing Chin, Batika M. J. Rana, Alexandros Englezakis, Rinako Nakagawa, Padraic G. Fallon, Anna Git, Andrew N. J. McKenzie

**Affiliations:** ^1^Medical Research Council Laboratory of Molecular Biology, Cambridge, United Kingdom; ^2^Department of Medicine, University of Cambridge, Cambridge, United Kingdom; ^3^Cambridge University Hospitals NHS Foundation Trust, Cambridge, United Kingdom; ^4^Immunity and Cancer Laboratory, Francis Crick Institute, London, United Kingdom; ^5^Trinity Biomedical Sciences Institute, Trinity College Dublin, Dublin, Ireland; ^6^Li Ka Shing Centre, Cancer Research UK Cambridge Institute, Cambridge, United Kingdom; ^7^Department of Biochemistry, University of Cambridge, Cambridge, United Kingdom

**Keywords:** miR-155, ILC2, apoptosis, IL-33, type-2 immunity

## Abstract

Group-2 innate lymphoid cells (ILC2) play critical roles in the initiation and maintenance of type-2 immune responses, predominantly through their production of the type-2 cytokines IL-5, IL-9, and IL-13. ILC2 are essential for the efficient elimination of helminth parasites, but also contribute to the detrimental type-2 immune responses that underlie diseases such as asthma and allergy. While several transcription factors have been identified that regulate the development and function of ILC2, less is known about the post-transcriptional mechanisms that regulate these processes. We identified micro-RNAs (miRNAs) that are co-ordinately regulated in ILC2 from mice exposed to two different stimuli, namely IL-33 “alarmin” administration or *Nippostrongylus brasiliensis* parasitic worm infection. miR-155 is upregulated in ILC2 in response to both stimuli and miR-155^−/−^ mice had impaired IL-33-driven ILC2 responses. Using mixed bone marrow chimeras, we demonstrate that this deficit is intrinsic to ILC2 and that miR-155 protects ILC2 from apoptosis, while having little impact on ILC2 proliferation or cytokine production. These data reveal a subset of miRNAs that are regulated upon ILC2 activation and establish a specific role for miR-155 in regulating ILC2 survival following activation.

## Introduction

Group-2 innate lymphoid cells (ILC2) are tissue-resident immune cells that represent a significant early source of the type-2 cytokines IL-5, IL-9, and IL-13 and play important roles in the initiation and maintenance of type-2 immune responses. ILC2 play critical roles in the elimination of helminth parasites (1), but also in the etiology of allergic diseases such as eczema (2) and asthma (3–5). ILC2 are activated by damage-associated signals, such as the epithelium-derived cytokines IL-25, IL-33 (6), and TSLP (7), which bind to the receptors IL-17RB, ST2 (IL-1RL1), and TSLP-R, respectively, on the ILC2 surface. In addition, ILC2 are activated by arachidonic acid metabolites such as PGD2, LTD_4_, and PGD_4_ (8), but inhibited by lipoxin A_4_ (9) and IFNγ (10).

ILC2 originate from common lymphoid progenitors (CLP) and several key transcription factors have been identified that regulate their development and function (11). Development of all ILCs requires the combined actions of several key factors, including Id2, Gata3, PLZF, and TCF-1 (12). Lineage-specific factors, such as RORα, Bcl11b, and high levels of Gata3, are subsequently required to specify the ILC2 lineage specifically.

Although there has been significant focus on the transcription factors that regulate ILC2 function, relatively little is known about the post-transcriptional mechanisms that govern ILC2 development and function. MicroRNAs (miRNAs) are important regulators of cell fate decisions and act to co-ordinately repress the expression of multiple genes in order to drive cellular re-programming. Indeed, miRNAs have been shown to play critical roles in Th2 cell development and function and perturbations in the expression of particular miRNAs are associated with allergy and asthma (13, 14). For example, miR-24 and miR-27 cooperate to suppress Th2 cell differentiation, serving to repress expression of the canonical Th2 transcription factor GATA3, as well as other critical Th2 cell regulators such as Ikzf1, and IL-4 in mice (15). Conversely, miRNA (miR)-19a promotes Th2 cell development through its repression of negative regulators of TCR signaling (*Pten*) and type-2 cytokine production (*Socs1* and *Tnfaip3*) and is reported to be upregulated in T cells from asthmatic airways (16). Recently, the miR-17~92 cluster that includes miR-19a was also found to promote IL-5 and IL-13 production from ILC2 (17).

A Th2 cell-intrinsic role has also been identified for miR-155, which is contained within exon 2 of the non-coding RNA, B cell integration cluster (bic). Inhibitors of miR-155 reduced IL-13 and IL-5 expression by Th2 cells, suggesting that miR-155 contributes to Th2 effector function, and miR-155-deficient mice exhibited significantly reduced airway inflammation and eosinophilia in response to house dust mite challenge or a model of ovalbumin-induced allergic airway inflammation (18, 19). A number of putative miR-155 targets have been identified in Th2 cells including genes encoding the sphingosine-1-phospate receptor 1 (S1PR1) (19), which may control lymphocyte egress from lymphoid tissue, the transcription factor PU.1 (18), a negative regulator of type-2 cytokine production, and metabolic regulators such as the mTOR binding partner, RICTOR (19). Recently, miR-155 has also been reported to regulate ILC2 and it was observed that miR-155-deficient mice have reduced numbers of ILC2 in response to ovalbumin-induced lung inflammation (20). Furthermore, ILC2 from *miR-155*^−/−^ mice exhibited impaired proliferation and IL-13 production in response to IL-33, though this study did not fully address whether this reflected an ILC2-intrinsic role for miR-155, or an effect of miR-155-dependent stromal or T cell-dependent factors (20). Indeed, allergen-induced IL-33 induction in the lung was significantly impaired in *miR-155*^−/−^ animals, suggesting pleiotropic roles for this miRNA (20).

To identify miRNAs that regulate ILC2 development and function, we performed comprehensive microarray analysis, with PCR verification, to identify miRNA expression in resting ILC2 from naïve mice, and activated ILC2 isolated from mice administered exogenous IL-33, or infected with the parasitic helminth *Nippostrongylus brasiliensis*. A subset of miRNAs was differentially expressed in both IL-33 and *N. brasiliensis* stimulated ILC2, while other miRNAs were regulated according to the particular stimulus used. Among those co-ordinately regulated by both IL-33 and *N. brasiliensis* infection were immunoregulatory miRNAs, including miR-155. Focussing on miR-155, due to its reported roles in type-2 immunity, we used mixed bone marrow (BM) chimeras and *in vitro* proliferation and survival assays to demonstrate that ILC2-intrinsic expression of miR-155 is required to protect ILC2 from apoptosis.

## Materials and methods

### Mice

*bic/miR155-deficient* mice (Mir155^tm1.1Brd^) (21) and *Rora*^fl/fl^ x *Il7ra*^Cre^ mice have been described previously (1) and CD45.1^+^ transgenic (B6SJL) mice were produced in-house. All mouse experiments were undertaken with the approval of the UK Home Office. Mice were kept at a specific pathogen-free facility.

### Murine models

Three murine models were used to study ILC2 responses in the presence or absence of miR-155. For IL-33 exposures mice were injected intraperitoneally with 1 μg of recombinant mouse IL-33 in PBS for 3 consecutive days. For IL-2/IL-25 expansion of ILC2 *in vivo*, mice were injected with 0.5 μg IL-2, 2.5 μg anti-IL2 and 0.5 μg of IL-25 in PBS for 3 consecutive days (1). Mice were euthanized on day 4. For *N. brasiliensis* infection, mice were administered 500 viable third-stage *N. brasiliensis* larvae subcutaneously and culled on day 5. Infection was confirmed by enumerating adult worm burden in the small intestine.

### Cell sorting and flow cytometry

In initial experiments to measure the miR response, abdominal (mesenteric, para-aortic, and inguinal) lymph nodes were harvested. For quantification experiments, mesenteric lymph nodes (MLN) from mice were homogenized and cell suspensions stained using fluorescent antibodies. For cell sorting, T-cells were defined as CD3^+^CD4^+^ cells and ILC2 were defined as Lin^−^ICOS^+^ cells. Lineage included CD3, 4, 5, 8, 11b, 11c, 19, B220, FceRI, NK1.1, and Ter-119. For flow cytometry staining (BD Fortessa) ILC2 were defined as live CD45^+^Lin^−^Sca1^+^ICOS^+^KLRG1^+^. For a complete list of antibodies used please see below.

### Antibodies

The following antibodies were used: FITC-conjugated anti-CD3 (Biolegend cat#100306, clone 145-2C11, RRID:AB_312671), FITC-conjugated anti-CD4 (BD, clone H129.19, Cat# 553651, RRID:AB_394971), FITC-conjugated anti-CD8 (eBioscience Clone 53.6.7, Cat# 11-0089-42, RRID:AB_10718971), FITC-conjugated anti-CD11b (Biolegend, clone M1/70 Cat# 101206, RRID:AB_312789), FITC-Conjugated anti-CD11c (eBioscience, clone N418, Cat# 11-0114-81, RRID:AB_464939), FITC-conjugated anti-CD19 (eBioscience Clone ID3 Cat# 11-0193-82, RRID:AB_657666), FITC-conjugated anti-B220 (eBioscience clone RA3-6B2 Cat# 11-0452-82, RRID:AB_465054), FITC-conjugated anti-FcERI (eBioscience clone MAR-1 Cat# 11-5898-81, RRID:AB_465307), FITC-conjugated anti-NK1.1 (BD, clone PK136 Cat# 553164, RRID:AB_394676), FITC-conjugated anti Ter119 (eBioscience clone Ter-119 Cat# 11-5921-81, RRID:AB_465310), APC-conjugated anti-ICOS (eBioscience clone C398.4A Cat# 17-9949-80, RRID:AB_11149500), Pe-conjugated anti-KLRG1 (eBioscience clone 2F1 Cat# 12-5893-80, RRID:AB_10597431), FITC-conjugated anti-ST2 (MD Bioscience Clone DJ8 Cat# 101001F, RRID:AB_947549), fixable viability dye R780 (eBioscience 65-065-14), BV510 conjugated anti-CD45.1 (BioLegend Clone A20 Cat# 110741, RRID:AB_2563378), AlexaFluor700-conjugated anti-CD45.2 (eBioscience clone 104 Cat# 11-0454-81, RRID:AB_465060), biotinylated anti-CD127 (Biolegend Clone SB/199 Cat# 135006, RRID:AB_2126118), PE-conjugated anti-Flt3l (eBioscience Clone A2F10 Cat# 15-1351-82, RRID:AB_494219), APC-conjuated anti-LPAM-1 (BioLegend clone Dakt32 Cat# 120608, RRID:AB_10730607), PeCF594-conjugated anti-CD25 (BD clone PC61), PeCy7-conjugated anti Sca-1 (eBioscience clone D7 Cat# 25-5981-82, RRID:AB_469669), PerCP-Cy5.5-conjugated anti-ICOS (BioLegend C398.A4 Cat# 313518, RRID:AB_10641280), FITC-conjugated anti-CD117 (BD clone 2BL Cat# 553354, RRID:AB_394805), BV510-conjugated anti-CD45 (BioLegend Clone 3F11 Cat# 103138, RRID:AB_256306), and eFlour450 conjugated anti-CD3, 4, 11b, 11c, 19, NK1.1, FcER1, F4/80, Ter119 (all eBioscience clones 145-2C11 Cat# 48-0031-80, RRID:AB_10733280, Gk1.1 Cat# 48-0041-82, RRID:AB_10718983, M1/70 Cat# 48-0112-82, RRID:AB_1582236, N418 Cat# 48-0114-82, RRID:AB_1548654, eBio103 Cat# 48-0193, RRID:AB_2043815), PK136 Cat# 48-5941-80, RRID:AB_2043878, Mar1 Cat# 48-5898-80, RRID:AB_2574085, BM8 Cat# 48-4801-80, RRID:AB_1548756 and Ter-119 Cat# 48-5921-82, RRID:AB_1518808, respectively), anti-IL-2 for injection (2Bscientific clone JES6-IAI2), PE-conjugated anti-Flt3 (E-bioscience clone A2F10 Cat# 15-1351-82, RRID:AB_494219).

### cDNA synthesis, qPCR, and microarray

RNA was isolated using trizol and the Qiagen RNeasy kits. Samples were analyzed using the Agilent RNA 6000 Pico Chip and 2100 Bioanalyser. cDNA for qPCR was synthesized using the Quantitec Reverse Transcription Kit and the Taqman microRNA assay (mmu-miR-155 and U6) according to the manufacturers' specifications.

### MicroRNA screen

ILC2 (CD3^−^CD4^−^Lin^−^ICOS^+^ cells) were isolated from naïve, IL-33 or *N. brasiliensis* treated mice using fluorescent cell sorting (Sony iCyt Synergy cell sorter) and stored after the addition of TRIzol LS. RNA was extracted using chloroform phase separation, followed by DNase treatment and further purification using Qiagen RNeasy micro kits (performing washes with 100% ethanol to preserve short RNA sequences). RNA integrity was checked using a RNA6000 Pico Kit on an Agilent 2100 Bioanalyzer. Labeling, microarray hybridization, washing, scanning and feature extraction were performed as described previously (22). As up to 150 mice had to be used to extract sufficient microRNA for microarray analysis of naïve mice, only a single measurement was taken due to cost and ethical considerations. Microarrays were scanned on an Agilent Microarray Scanner (G2565CA) using miRNA_107_Sep09 scanning protocol (Agilent). Spot intensities were then extracted using Agilent's Feature Extraction software v10.7.3. Microarray results were compared to qPCR, obtained using Exiqon's miRCURY LNA Universal RT microRNA PCR Mouse&Rat plates I and II V3.M.

### Retroviral construct preparation

A pSiren-RetroQ retroviral construct (Clontech) was modified by replacing the puromycin resistance gene with an mCherry-expressing gene. Two double stranded oligonucleotides (5′-GATCCACCCCTATCACAATTAGCATTAATTCAAG AGATTAAT GCTAATTGTGATAGGGGTTTTTTTG−3′, 5′-AATTCAAAAAAAC CCCTATCACAATTAGCATTAATCTCTTGAATTAATGCTAATTGTGATAGGGGTG−3′, controls 5′-GAT CCGATTATGTGAGAGCGTGTATTATTTCAAGAGAATAATACACGCTCTCACATAATCTTTTTACGCGTG-3′, 5′-AATTCACGCGTAAAAAAGATTATGTGAGAGCGTGTAT TATTCTCTTGAATAATACACGCTCTCACATAATCG-3′) with homology to the entire region of mmu-miR-155-5p and containing BamHI/EcoRI overhangs and an internal hybridisation region, were annealed and ligated downstream of the U6 promoter of the pSiren-retroQ mCherry retroviral vector.

### Construction and collection of the recombinant retrovirus

HEK-293T cells maintained in DMEM/10%FCS/penicillin and streptomycin were co-transfected (5 × 10^6^ cells) using polyethylenimine with 10 μg of anti-miR-155 pSiren-retroQ and 15–20 μg of the ecotrophic packaging vector pCL-Eco (Imgenex) and the cells were incubated at 37°C. After 72 h, the supernatant containing the retroviral particles was collected and filtered through a low protein-binding filter (0.45 μm). The supernatant was centrifuged at 25 k rpm at 4°C for 2 h using an ultracentrifuge and the supernatant removed. The virus particles were resuspended in a primary cell culture compatible media (RPMI, 10% FCS, P/S) for a 20x concentration and stored at −80°C.

### ILC2 transduction

MLN from IL-2/anti-IL-2/IL-25 stimulated mice were collected and pooled. The mouse tissue cell supsension was then depleted of lineage positive cells using biotinylated antibodies (B220, CD3e, CD4, CD5, CD8, CD11b, CD11c, FceRIa, Gr-1, NK 1.1, TER119) and Dynabeads (Thermo Fisher) for magnetic separation. Lineage negative cells were cultured for 24 h with IL-33 (10 ng/ml) and IL-7 (10 ng/ml). 2 × 10^5^ ILC2 were seeded per well and transduced with viral supernatant. The cells were then centrifuged at 900 × g, at 32°C for 90 min and incubated in the presence of IL-33 (10 ng/ml) and IL-7 (10 ng/ml). After 72 h, transduced cells (Live, Lineage^−^ICOS^+^KLRG1^+^mCherry^+^) were sorted using an iCyt Synergy cell sorter (Sony). Isolated ILC2 were cultured with IL-33 (10 ng/ml) and IL-7 (10 ng/ml). Cells were counted every day for 5 days.

### Apoptosis

JC-1 staining was performed according to the manufacturers (ABCAM) instructions. AnnexinV staining was performed using eBioscience PerCP-efluor710 labeled AnnexinV and a fixable viability dye. As controls, mitochondria were depolarized using FCCP or cell death induced using heat shock.

### Statistical analysis

Spot intensities from the Agilent Microarray were obtained with background correction and gProcessedSignal data were analyzed. The minimum measure of variability was calculated and added to each value to remove negative values and data was Log2 transformed. Principal component analysis was performed as well as a two-way ANOVA to assess miRNA fold changes and generate hierarchical clustering heat maps. All miRNA analysis was performed using Partek® Genomics Suite® software, version 7.17 Copyright; 2017. Other data were analyzed using Graphpad Prism using *t-*tests or ANOVA, as indicated for individual experiments.

## Results

### Differential regulation of miRNAs in ILC2 following activation

**Figure 1:**
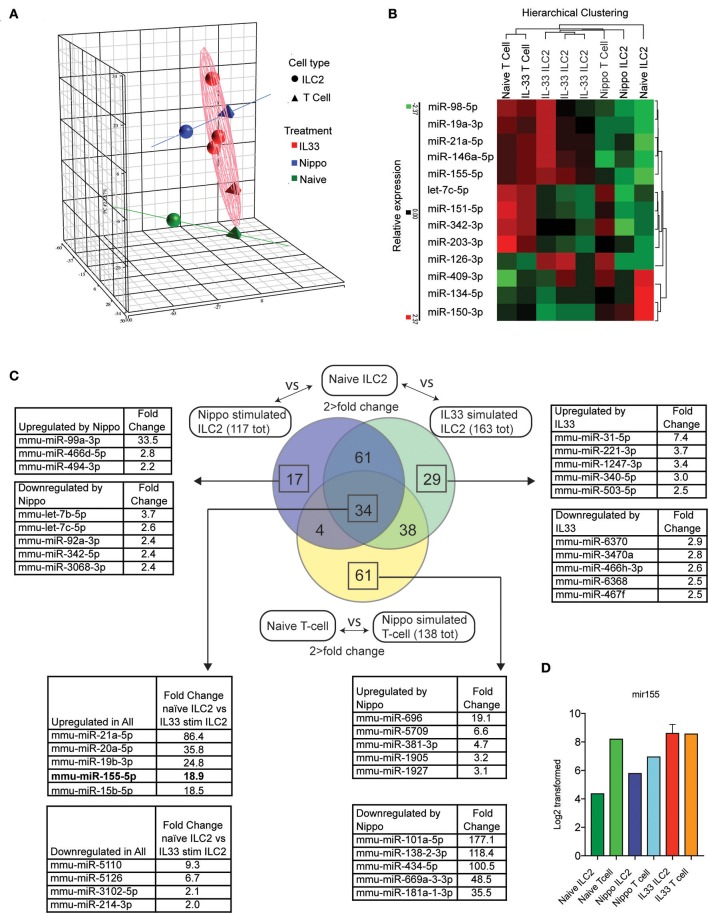
Differential regulation of miRNAs in ILC2 following activation. **(A)** PCA analysis of microRNA expression by ILC2 from mice stimulated with IL-33 or infected with *N. brasiliensis* (5 d.p.i,). Tetrahedrons represent T cells, spheres represent ILC2, red for IL-33 stimulation, blue for *N. brasiliensis* exposure and green for naïve cells. Ellipsoid and colored lines show the relationship between stimulation type and miRNA variation. **(B)** Relative expression and hierarchical clustering of miRNA known to be involved with leukocyte activity, relative expression is indicated (red, higher; green, lower). **(C)** Venn diagram of miRNAs that exhibit a >2-fold change in expression in IL33 or *N. brasiliensis* stimulated ILC2 compared with naïve ILC2 and similarly for T cells exposed to *N. brasiliensis* compared with naive T cells. Lists of miRNA with the top 5 greatest fold-change values, both upregulated and downregulated, are shown for the indicated sections where applicable. The list corresponding to the 34 miRNA changed in all 3 populations show the top 5 upregulated and downregulated miRNA in IL33 stimulated compared with naïve ILC2s are shown. **(D)** MiR155 log2 transformed data are shown for each sample.

To determine the expression of miRNAs in ILC2 following activation, we isolated ILC2 (CD3^−^CD4^−^Lineage^−^ICOS^+^) from the abdominal lymph nodes of naïve wildtype (WT) mice or WT mice treated with IL-33 or infected with *N. brasiliensis* (day 5 post-infection). miRNA expression profiles were established using microarray and confirmatory PCR (IL-33 data only, data not shown). Principle component analysis (PCA) is shown in Figure 1A. Many miRNA already identified to be involved with lymphocyte activity were detected in this data set and their relative expression between stimulated and naive samples are shown (Figure 1B). Data revealed 95 miRNAs in ILC2 that were differentially regulated in response to both IL-33 and *N. brasiliensis*, with a further 67 unique to IL-33 and 21 unique to *N. brasiliensis* (Figure 1C, Supplementary Tables 1–7). Of these, miR-155 was selected for further study as it ranked highly in fold-change (18.9-fold increase) in IL-33-treated mice and has been implicated previously in lymphocyte development and Th2 cell immunity. miRNA differentially expressed between naïve ILC2 and ILC2 following *in vivo* IL33-stimulation or *N. brasiliensis*-infection, and T cells upon *N. brasiliensis*-infection indicated considerable co-incidence of miRNA modulation (Figure 1C and Supplementary Tables 1–7). Notably 34 miRNA were found to be changed in all stimulated populations (Figure 1C). These 34 miRNAs included mir-155, mir-146a, and mir-19a (Figure 1C), of which mir-155 was among the most highly expressed in activated cells; intensity data for miR155 are shown for each sample (Figure 1D).

### miR-155 is required for ILC2 expansion response to IL-25 or IL-33

**Figure 2:**
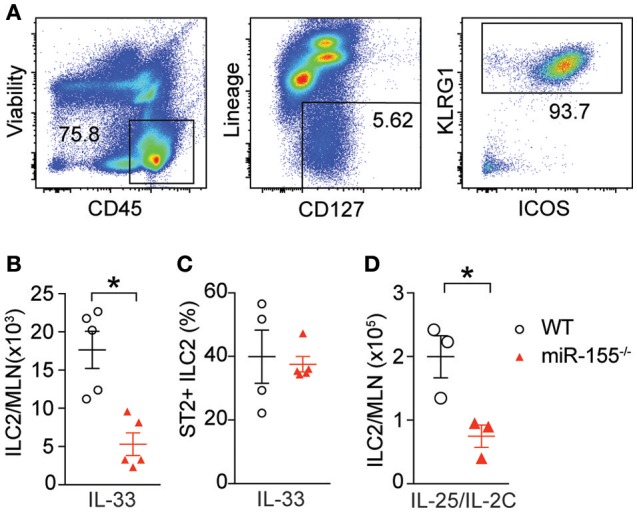
miR-155 is required for ILC2 expansion in response to IL-25 and IL-33. WT and *miR155*^−/−^ mice were treated with intra-peritoneal (i.p.) IL-33 **(A–C)** or IL-2/anti-IL-2/IL-25 complex **(D)**, for 3 days. **(A)** Representative staining of ILC2 (CD45^+^Lin^−^ICOS^+^KLRG1^+^) isolated from mesenteric lymph node (MLN). **(B)** Quantification of ILC2 in MLN. **(C)** Percentage ST2+ WT and *miR155*^−/−^ ILC2. **(D)** Quantification of ILC2 following IL-2/anti-IL-2/IL-25 (IL-25/IL-2C) complex treatment. Data **(A,B,D)** are representative of three independent experiments; **(C)** a single experiment. *p ≤ 0.05 with Student's *t-*test.

To study the effect of miR-155 on ILC2 we injected both wildtype (WT) and *miR155*^−/−^ mice with IL-33 to expand ILC2 *in vivo*. ILC2 were isolated from the mesenteric lymph nodes (MLN) and enumerated. As expected, IL-33 increased ILC2 numbers in WT mice. However, there were significantly fewer ILC2 in the MLN of miR-155-deficient mice treated with IL-33 (Figures 2A,B). There were no differences in the expression of ST2 (the IL-33 receptor) between WT and *miR155*^−/−^ ILC2 (Figure 2C). Furthermore, although administration of IL-25 in combination with a complex of IL-2 and anti-IL2 (to maintain the slow release of IL-2 and maximize ILC2 proliferation) also induced an expansion in ILC2 numbers in WT mice, this increase was reduced in the absence of miR-155 (Figure 2D). Thus, miR-155 is required for the increase in ILC2 numbers in response to IL-25 or IL-33 stimulation.

### Haematopoetic cell miR-155 is critical for ILC2 expansion in response to IL-33

**Figure 3:**
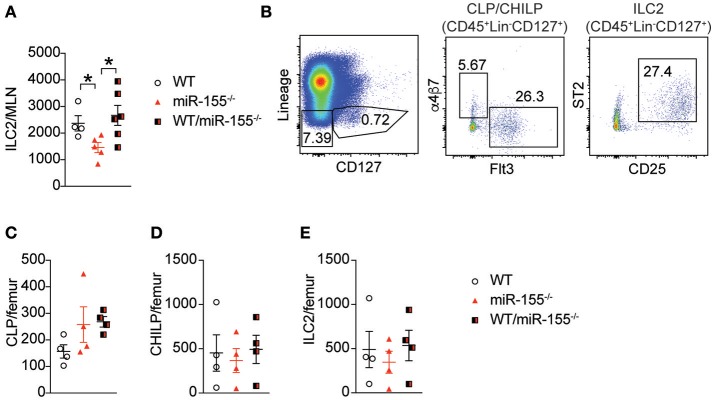
Haematopoetic cell miR-155 is critical for ILC2 expansion. Lethally irradiated B6SJL mice were reconstituted with WT, *miR155*^−/−^ or mixed WT/*miR155*^−/−^ BM, and treated with IL-33 for 3 days. **(A)** Numbers of ILC2 (live CD45^+^Lin-CD127^+^Sca1^+^KLRG1^+^ICOS^+^) from MLN. **(B)** Representative staining of BM progenitor cell populations. **(C)** Enumeration of BM-derived common lymphoid precursors (CLP: Lin^−^CD127^+^Flt3^+^), **(D)** common helper innate lymphoid precursors (CHILP: Lin^−^CD127^+^Flt3-a4b7^+^CD25^−^), and **(E)** ILC2 precursors (Lin^−^CD127^+^CD25^+^ST2^+^). Data are representative of two independent experiments, *n* = 4–7. *p ≤ 0.05 with Student's *t-*test.

Early studies suggested that miR-155 is only expressed in haematopoetic cells (23). However, subsequent studies have reported expression of miR-155 in other cell types, such as endothelial cells (24, 25), epithelial cells (26, 27) and fibroblasts (28). To exclude potential miR-155-related perturbation of epithelium-derived cytokines or other stromal cell factors, we generated bone marrow (BM) chimeric mice to study the effects of miR-155 within the haematopoietic compartment. Lethally irradiated WT CD45.1^+^ (B6SJL) mice received CD45.2^+^ BM from WT mice, miR-155-deficient mice, or an equal mix of WT and *miR155*^−/−^ BM cells. Following a period of 3 months (to allow reconstitution of donor-derived ILC2 populations), mice were treated with intra-peritoneal IL-33 and the number of donor-derived ILC2 enumerated. In mice that had received *miR155*^−/−^ BM only, there were fewer ILC2 in the MLN compared to mice that had received WT BM (Figure 3A). There were no differences between WT and mixed WT/*miR155*^−/−^ BM transplanted mice, indicating that the absence of miR-155 does not have a trans-inhibitory effect. Thus, the deficit in ILC2 numbers in miR-155-deficient mice is dependent on haematopoietic lineages, but not stromal cell signals.

### miR-155 does not regulate numbers of ILC2 precursors

To determine if miR-155 affected the expansion of ILC2 precursors, we studied the levels of common lymphoid progenitors (CLP), common helper innate lymphoid progenitors (CHILP) and ILC2 in the BM of IL-33-treated chimeric mice. There were no significant differences in BM CLP (Figures 3B,C), CHILP (Figure 3D), or ILC2 (Figure 3E) Thus, deficits in progenitor and precursor populations do not underlie the reduction in ILC2 numbers observed in the lymph nodes following IL-33 treatment.

### miR-155 increases ILC2 numbers through an ILC2 intrinsic mechanism

**Figure 4:**
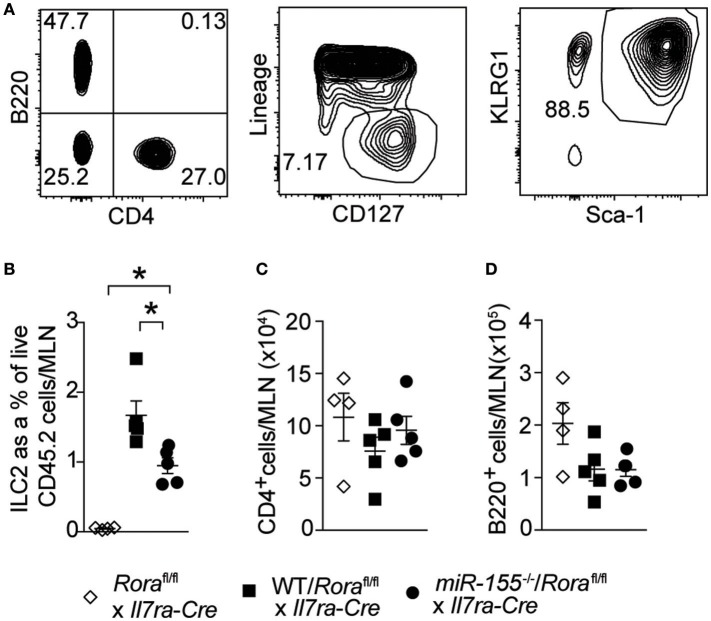
ILC2 intrinsic miR-155 is critical for ILC2 expansion. B6SJL mice were lethally irradiated and received BM from *Rora*^flox/flox^ x *Il7ra*^Cre^ mice alone or in equal proportions with WT or *miR155*^−/−^ BM. After 3 months to allow reconstitution, mice were treated with i.p. IL-33 (1 ug/dose) for 3 days. **(A)** Representative flow cytometry gating for the indicated populations. **(B)** Frequency of ILC2 (Lin^−^CD127^+^KLRG1^+^Sca1^+^) from MLN. Numbers of CD4^+^ T cells **(C)** and B cells **(D)** from MLN. Data are representative of three independent experiments. *p ≤ 0.05 with ANOVA with Tukey correction.

To establish whether the effect of miR-155 was intrinsic to ILC2 themselves, and not the actions of miR-155 in other haematopoetic cells (such as T cells) or indeed miR-155 containing exosomes derived from other haematopoetic cells (29, 30), we generated mixed WT/ILC2-deficient or *miR155*^−/−^/ILC2-deficient BM chimeric mice. ILC2-deficient BM was obtained from *Rora*^flox/flox^ x *Il7ra*^Cre^ mice (1). In these mice, the essential ILC2 transcription factor RORα is deleted in cells expressing the IL-7 receptor. This results in a depletion of ILC2, while there are no reported defects in T helper cell function (1, 31). Lethally irradiated WT CD45.1^+^ (B6SJL) recipient mice received either mixed equal proportions of WT/ILC2-deficient, or *miR155*^−/−^/ILC2 deficient BM. In these mice, ILC2 are derived from either WT or *miR155*^−/−^ cells, but other cells (stromal as well as at least a proportion of the haematopoetic cells) have unperturbed miR-155 signaling. While WT ILC2 expanded robustly in response to IL-33, this expansion was diminished in the absence of ILC2 intrinsic miR-155 (Figures 4A,B). There were no differences in MLN T-cell (CD4^+^–Figure 4C) and B-cells (B220^+^–Figure 4D) in the presence or absence of miR-155. These results support an ILC2-intrinsic role for miR-155 in regulating ILC2 expansion in response to IL-33.

### miR-155 is required for ILC2 expansion *in vitro*

**Figure 5:**
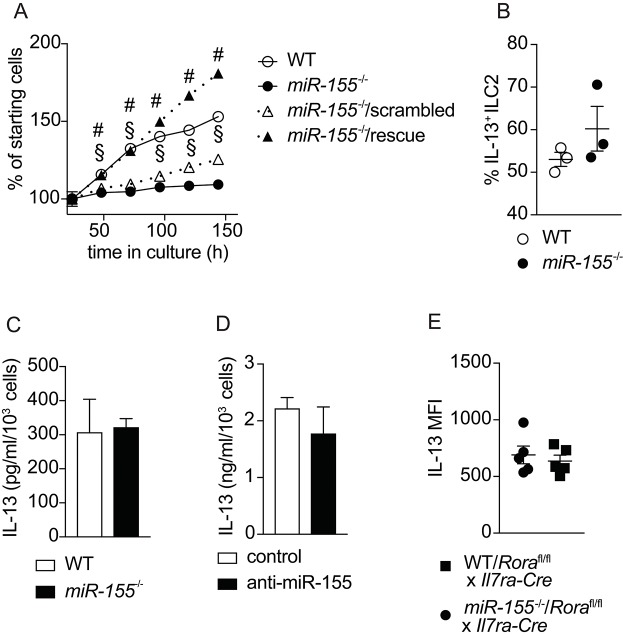
miR-155 is required for ILC2 expansion *in vitro* but not IL-13 production. ILC2 were isolated from MLN of IL-2/anti-IL-2/IL-25 treated WT or *miR155*^−/−^ mice. ILC2 were either left untransduced or transduced with a retrovirus over-expressing the miR-155 sequence to restore miR-155 signaling. Cells were cultured for 6 days in the presence of IL-7 and IL-33 (both at 10 ng/ml). **(A)** Daily ILC2 counts. **(B)** Intracellular IL-13 staining of WT or *miR155*^−/−^ ILC2 from 5-day cultures in **(A)** analyzed by flow cytometry. **(C)** Secreted IL-13 was quantified in supernatants from the cultures of WT and *miR155*^−/−^ ILC2 using ELISA (data corrected for cell counts). **(D)** Secreted IL-13 was quantified in supernatants from WT ILC2 treated with negative control siRNA or miR-155 knockdown siRNA harvested after 2 days using ELISA (data corrected for cell counts). **(E)** Intracellular IL-13 expression in ILC2 isolated from the MLN of IL-33-treated lethally-irradiated B6SJL mice that had received mixed BM from *Rora*^flox/flox^ x *Il7ra*^Cre^ mice with WT or *miR155*^−/−^ BM. Cells were treated with PMA/ionomycin in the presence of protein transport inhibitor. Data are representative of at least two independent experiments. Two-way ANOVA with Sidak's multiple comparisons test **(A)**, ^#^*p* ≤ 0.05 compared to *miR155*^−/−^, ^§^*p* ≤ 0.05 compared to control transduced miR-155^−/−^ ILC2.

To confirm the effects of miR-155 on the expansion of ILC2 that we observed *in vivo*, we harvested ILC2 from the mesenteric lymph nodes (MLN) of mice treated with IL-2/anti-IL-2/IL-25. Flow cytometrically purified ILC2 were expanded in culture with IL-7 and IL-33, and cell counts performed at regular intervals. As suggested by the *in vivo* data, we observed lower numbers of ILC2 in the absence of miR-155 (Figure 5A). To substantiate that this effect was miR-155-specific we transduced miR-155-deficient ILC2 with a retroviral vector over-expressing miR-155 (rescue) or a negative control sequence. Retroviral transduction of miR-155-deficient ILC2 with the negative control miRNA failed to restore ILC2 number to that of control cultures (Figure 5A). By contrast, following retroviral transduction with a vector over-expressing miR-155, *miR155*^−/−^ cells numbers were restored to the level of WT ILC2 (Figure 5A). These findings indicate that the effect of miR-155 in enhancing ILC2 numbers *in vivo* is replicated *in vitro* and specific to miR-155.

### miR-155 does not affect ILC2 cytokine expression

A previous publication reported effects of miR-155 on cytokine production by ILC2 (20). Hence, we examined whether miR-155 regulated IL-13 production by ILC2. After 5 days of culture with IL-7 and IL-33, there were no differences in the percentage of cells staining positive for intracellular IL-13 in the presence or absence of miR-155 (Figure 5B). In addition, we quantified the levels of IL-13 in supernatants collected from cultured WT and *miR155*^−/−^ ILC2 (Figure 5C), and ILC2 transduced with miR-155 knock-down vector (or negative control) using ELISA (Figure 5D). No differences in the levels of IL-13 were detected in the supernatants. We also quantified intracellular IL-13 expression by *ex vivo* ILC2 obtained from WT/ILC2-deficient, or *miR155*^−/−^/ILC2-deficient chimeric mice treated intra-peritoneally with IL-33. No differences were found in the expression of IL-13 in the presence or absence of miR-155 (Figure 5E). Taken together these results suggest that miR-155 does not directly regulate IL-13 production by ILC2.

### miR-155 protects activated ILC2 from apoptosis

**Figure 6:**
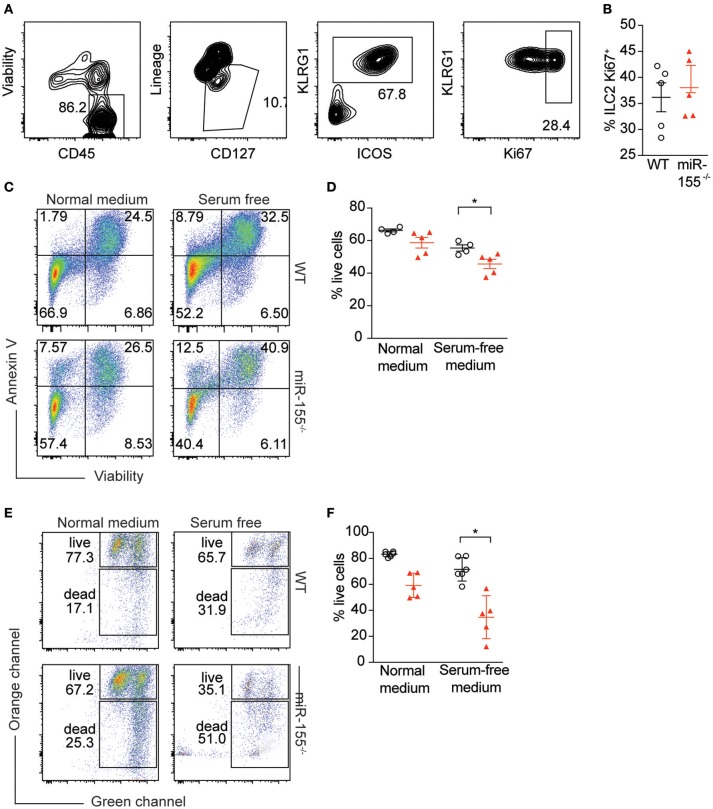
miR-155 protects ILC2 from undergoing apoptosis. LNs were excised from IL-33-treated WT and *miR155*^−/−^ mice and CD3^+^, B220^+^, CD11b^+^, and CD11c^+^ cells depleted using dynabeads. The remaining cells were cultured in 10 ng/ml IL-7 and IL-33 for 1 week and ILC2 proliferation assessed using Ki67 staining. **(A)** Representative flow cytometry for Ki67 staining. **(B)** Proportion of Ki67-positive ILC2 from LN analyzed *ex vivo* from WT and *miR155*^−/−^ mice treated *in vivo* with IL-33 for 3 days. **(C,D)** Annexin V and viability staining of ILC2. WT or *miR155*^−/−^ ILC2 were isolated from MLN of IL-2/anti-IL-2/IL-25- or IL-33- treated animals and then expanded *in vitro* in the presence of IL-33 for 5 days. Following culture, cells were either kept in growth medium or serum-starved (in RPMI only) for 6 h. **(C)** Representative FACS plots. **(D)** Enumeration of live vs. apoptotic cells. **(E,F)** Mitochondrial depolarisation was assessed using JC-1 staining (culture as for **C,D**). **(E)** Representative FACS plots. **(F)** Enumeration of live vs. apoptotic cells. Data are representative of two **(B–D)** or three **(E,F)** independent experiments. Student's *t-*test, *p ≤ 0.05.

To further study the mechanism by which miR-155 increases numbers of ILC2, we assessed potential differences in proliferation between WT and *miR155*^−/−^ ILC2. We enriched ILC2 by negative selection from lymph nodes of IL-33-stimulated mice, and after 1 week in culture with IL-7 and IL-33, ILC2 proliferation was assessed using Ki67 staining. We detected no differences in the proportion of Ki67^+^ ILC2 harvested from WT and *miR155*^−/−^ mice and cultured *in vitro* (Figures 6A,B).

Since we were unable to obtain evidence for a role for miR-155 in supporting ILC2 proliferation, we assessed whether the observed reduction in *miR155*^−/−^ ILC2 numbers compared to WT might be explained by increased ILC2 cell death in the absence of miR-155. WT and *miR155*^−/−^ ILC2 were expanded in IL-7 and IL-33. Cells were then either kept in growth medium, or apoptosis was induced using serum starvation for 6 h. We stained ILC2 using Annexin V (to detect externalization of membrane phosphatidylserine as a facet of apoptosis) and viability staining. Even under normal ILC2 culture conditions the absence of miR-155 resulted in more apoptotic cells in the *miR155*^−/−^ ILC2 cultures (Figures 6C,D). Furthermore, serum starvation for 6 h potentiated these differences (Figures 6C,D).

To confirm these findings, we assessed mitochondrial outer membrane potential (as measured by JC-1 staining), as an alternative indicator of apoptosis. While there was a trend toward an increase in apoptosis in *miR155*^−/−^ ILC2 cultures in complete medium as compared to WT controls (Figures 6E,F), this increase in apoptosis reached statistical significance after 6 h of serum starvation (Figures 6E,F). Although we also investigated the presence of markers of apoptosis directly *ex vivo*, we found there were very few apoptotic ILC2 in the LN of IL-33-treated mice, likely because apoptotic cells are rapidly cleared in the tissue. Together, our data indicate that the expression of miR-155 protects ILC2 from apoptosis during activation.

## Discussion

ILC2 are key players in type-2 immunity, and an understanding of their regulation and biology offers an opportunity for the development of new therapeutic targets in type-2 immune-mediated diseases such as asthma and allergy. Using comprehensive miRNA microarray and RT-qPCR strategies we identified a group of miRNAs whose expression was regulated in response to ILC2 activation *in vivo*, following either IL-33 treatment or *N. brasiliensis* infection, compared to naïve controls. Additional miRNAs were also regulated uniquely in response to IL-33 administration or worm infection, respectively. Of the shared miRNAs several have been associated with the regulation of cell growth and proliferation e.g. miR-93 (32, 33), miR-25 (34), miR-103 (35). Notably, others are known to regulate immune function, including miR-155, mir-146a and let-7a and let-7d (36). Furthermore, our data show good concordance with the ILC2 miRNA expression reported recently by Singh et al. using small RNA-sequencing (17). They went on to show that ILC2 deficient in the miR-17~92 cluster were less proliferative than wildtype controls, though the rate of apoptosis was not affected. miRNA-19a, located in the miR-17~92 cluster was also required for optimal IL-5 and IL-13 production by ILC2, and this contributed to allergic inflammation (17).

It has been reported recently that both miR-155 and miR-146a are required for the type-2 response to helminth infection. MiR-146a-deficient mice are unable to clear *Trichuris muris* infection, associated with a decrease in IL-13-producing T cells and an increase in IL-17-secreting T cells (19). *Heligmosomoides polygyrus* infection of miR-146a-deficient mice led to a decrease in the proportion of IL-5-producing T cells with an increase in IL-17 and IFN-γ producing T cells, suggesting that miR-146a was responsible for polarizing T cells to a Th2 profile. Furthermore, miR-155-deficient mice were also unable to mount a Th2 response when infected with *H. polygyrus* and miR-155 was upregulated in Th2 cells as well as the cells around the intestinal lesions during an *H. polygyrus* infection (19). MiR-155-deficient mice also had significantly lower IL-13 expression and airway mucus in response to house dust mite challenge (19), and impaired responses to an OVA-model of allergic airway inflammation (18). Additionally, miR-155 has been shown to play key roles in other cell types and immune responses. In the absence of miR-155, mice fail to produce high affinity IgG1 and have smaller germinal centers (37–39). MiR-155 is also crucial in Th17 cells (40), and mice deficient in miR-155 are resistant to experimental autoimmune encephalitis due to reduced production of Th17 and Th1 responses (41). Furthermore, in the absence of miR-155, CD8 T-cell responses against viruses and tumors are impaired (42). Finally, a recent report showed that ILC2 proliferate less readily in the absence of miR-155, however, an ILC2-intrinsic requirement for miR-155 *in vivo* was not explored and the mechanism by which miR-155 increased ILC2 numbers was not determined (20).

We confirmed that there are fewer ILC2 in the lymph nodes of IL-33-treated miR-155-deficient mice compared to WT controls, and that this is due to an ILC2-intrinsic role for miR-155 in maintaining ILC2 numbers during immune activation by protecting them from apoptosis. While miR-155 is widely expressed in haematopoetic cells, several reports indicate expression in stromal tissues, including epithelial cells. Generating BM chimeric mice allowed us to demonstrate that the effects of miR-155 on ILC2 were dependent on the haematopoetic compartment. Furthermore, by generating mixed BM chimeras using miR155-deficient BM and ILC2-deficient BM from *Rora*^flox/flox^ x *Il7ra*^Cre^ mice, we established that the effect of miR-155 on ILC2 numbers is intrinsic to ILC2. This effect was recapitulated *in vitro*, with fewer *miR155*^−/−^ ILC2 present after culture with IL-7 and IL-33 as compared to WT ILC2, and this was rescued by retroviral over-expression of miR155.

Interestingly, there were no differences in BM ILC2 precursors, such as haematopoetic stem cells, CLP or CHILP in the presence or absence of miR-155. Thus, miR-155 did not appear to regulate ILC2 numbers *in vivo* by increasing proliferation of ILC2 precursors. Also, there were no differences in BM ILC2. Hence, it is unlikely that the increased numbers of ILC2 in peritoneal lymph nodes were caused by increased ILC2 progenitor production.

Notably, although others have reported a defect in miR-155-deficient ILC2 proliferation (20), we did not observe differences in the presence of Ki67^+^ ILC2 cultured *in vitro* or when analyzed *ex vivo*, as compared to WT controls. In contrast to Johansson et al. who assessed the percentage of total Ki67^+^Lin^−^ cells compared to total lung cells (which does not take into account the differences in ILC2 numbers that are present in the absence of miR-155), we specifically determined the proportion of proliferating ILC2 compared to total ILC2 to account for the differences in total ILC2 numbers in the *miR155*^−/−^ mice. Furthermore, as others have also reported effects of miR-155 on cytokine production (21), and indeed on the production of IL-13 by ILC2 (20), we measured IL-13 production by ILC2 in the presence or absence of miR-155. However, we did not find any differences in the ability of ILC2 to produce IL-13 in the presence or absence of miR-155, as measured by ELISA and intracellular cytokine staining. It is not clear why these data differ, though the mouse lines used in the different studies, or the facilities, could contribute. Our study employed the mir-155-deficient mouse strain (Mir155^tm1.1Brd^) generated by Bradley and colleagues (21), whilst Johanssen et al. used the *miR155*^−/−^ mice (Mir155^tm1.1Rsky^) from Rajewsky and colleagues (38). Both groups targeted exon 2 in 129Sv-derived embryonic stem cells, removed the neomycin selection cassette, and backcrossed to a C57BL/6 background. However, the Mir155^tm1.1Rsky^ line also retains a LacZ reporter. An alternative explanation for the differences observed is that our study and that of Johannson et al. investigated ILC2 from different tissues; lymph nodes or lungs, respectively. Consequently, variances may arise from the kinetics of the response and the time-points analyzed.

Since we had confirmed that the effects of miR-155 on ILC2 numbers were preserved in response to IL-25, and that miR-155 did not alter ST2 levels, it is unlikely that miR-155 interferes with IL-33 receptor signaling specifically. However, using two different methods to quantify apoptosis (JC-1 staining as a measure of loss of mitochondrial membrane potential, and annexin V), we identified that miR-155 expression protects stressed ILC2 from undergoing apoptosis, thereby maintaining and increasing ILC2 numbers. Previous studies have linked miR-155 to apoptosis, both in pro- and anti-apoptotic roles. MiR-155 has also been shown to be pro-apoptotic in acute myeloid leukemia (43) and human dendritic cells (44). However, extensive evidence links miR-155 with anti-apoptotic functions, including in immune cells. In pancreatic cancer ductal cells, miR-155 targets tumor protein 53-induced nuclear protein 1, resulting in decreased apoptosis and increased tumorogenic potential (45). Others have suggested that miR-155 targets caspase-3 and Fas-associated protein with death domain (FADD) directly to protect cells from apoptosis (46). In psoriasis, miR-155 is thought to result in increased proliferation and reduced apoptosis by targeting the PTEN signaling pathway (47). FOXO3 is a target of miR-155, and miR-155 inhibits apoptosis by targeting FOXO3 in monocytes (48) and T cells (49). In B cells, JARID2 is targeted by miR-155, inhibiting apoptosis and resulting in germinal center B cell expansion through collaboration with c-Myc (50). To our knowledge our data are the first linking miR-155 to the control of apoptosis in innate lymphoid cells. Interestingly, recent studies have also indicated that miR-155 promotes autophagy to protect cells during stresses such as hypoxia and nutrient deprivation (51, 52). Thus, it is possible that in the absence of miR-155 ILC2 have impaired autophagy and that this contributes to their decreased survival. However, further studies will be required to identify miR155 target genes in the regulation of apoptosis in ILC2, and to investigate potential links between miR155 and autophagy-associated cell death (53).

In summary, we present a comprehensive analysis of miRNA changes in ILC2 in response to activation and demonstrate that miR-155 increases ILC2 frequency in response to stimulation by reducing apoptosis. Although we were unable to identify the target(s) of miR-155 that orchestrate control of apoptosis in ILC2, our data provide a novel pathway for the regulation of ILC2 through apoptosis.

## Author contributions

MK, SC, AE, and AG designed and performed experiments, analyzed data, and made figures. BR analyzed data. RN and PF provided reagents. AM conceived the study and designed experiments. All authors contributed to writing the paper.

### Conflict of interest statement

AM has grant funding from GSK and AstraZeneca/MedImmune. The remaining authors declare that the research was conducted in the absence of any commercial or financial relationships that could be construed as a potential conflict of interest.
